# Analysis of cGMP signalling with transgenic mice expressing FRET-based cGMP sensors

**DOI:** 10.1186/2050-6511-14-S1-P76

**Published:** 2013-08-29

**Authors:** Lai Wen, Martin Thunemann, Susanne Feil, Matthias Hillenbrand, Angelos Vachaviolos, Thomas Ott, Xiaoxing Han, Dai Fukumura, Rakesh K Jain, Cor de Wit, Michael Russwurm, Robert Feil

**Affiliations:** 1 Interfakultäres Institut für Biochemie, University of Tübingen, Tübingen, Germany; 2 IZKF - Transgene Tiere, Universität Tübingen, Tübingen, Germany; 3 Edwin L. Steele Laboratory for Tumor Biology, Department of Radiation Oncology, Massachusetts General Hospital (MGH) and Harvard Medical School, Boston, Massachusetts, USA; 4 Institut für Physiologie, Universität zu Lübeck, Lübeck, Germany; 5 Institut für Pharmakologie und Toxikologie, Universität Bochum, Bochum, Germany

## Background

Sensor proteins that detect cGMP are valuable tools to analyse the molecular mechanisms underlying cGMP’s manifold physiological functions. These cGMP sensors allow for visualization and quantification of cGMP in living cells, in real time, and at high spatial and temporal resolution. For instance, they allow one to study sub-cellular cGMP compartments, and combined with multi-photon microscopy, they can provide new insights into complex biological processes, which can be analysed only in live animals.

## Results

To monitor cGMP signals in vivo, we have generated and characterized transgenic mouse lines expressing the fluorescence resonance energy transfer (FRET)-based cGi500 sensor (cGMP indicator with an EC_50_ value for cGMP of 500 nM, [1]). One mouse line produced by random transgenesis expresses cGi500 in vascular and visceral smooth muscle. Other mouse lines were created by targeted modification of the ROSA26 locus; these lines either show ubiquitous cGi500 expression, or they can be used for Cre/lox-dependent, tissue-specific cGi500 expression. These cGi500-transgenic mice are healthy and fertile, and do not show obvious adverse phenotypes.

Primary cells isolated from cGi500-expressing mice, including platelets, neural cells, and smooth muscle cells were used for FRET imaging experiments. NO-induced cGMP synthesis via soluble guanylyl cyclase was observed in the majority of cell types, while cGMP responses to atrial or C-type natriuretic peptides differed between cell types. Moreover, NO-induced cGMP elevations could be observed in the vasculature of retinas isolated from cGi500-expressing animals. Importantly, the feasibility of FRET-based cGMP imaging in living animals was demonstrated by intravital microscopy of anesthetized cGi500-transgenic mice. By epifluorescence FRET microscopy we could visualize transient NO-induced cGMP signals in arterial walls of the cremaster muscle. Furthermore, we used multi-photon microscopy in a dorsal skinfold chamber model and were able to record NO-elicited FRET changes in subcutaneous vessels.

## Conclusion

All in all, these cGMP sensor mouse lines represent valuable resources for studies with cGi500-expressing primary cells, and, importantly, they can also be used for intravital imaging studies with live animals. We believe that these mouse lines will find widespread applications and that in combination with other genetic mouse models they can contribute to improve our understanding of cGMP-modulated physiological or disease-associated processes.

## References

[B1] Russwurm M Mullershausen F Friebe A Jager R Russwurm C Koesling D Design of fluorescence resonance energy transfer (FRET)-based cGMP indicators: a systematic approach Biochem J 2007 407 69 77 10.1042/BJ20070348 17516914 PMC2267402

